# Sounding out life in the deep using acoustic data from ships of opportunity

**DOI:** 10.1038/s41597-020-00785-8

**Published:** 2021-01-20

**Authors:** K. Haris, Rudy J. Kloser, Tim E. Ryan, Ryan A. Downie, Gordon Keith, Amy W. Nau

**Affiliations:** CSIRO Oceans and Atmosphere, GPO Box 1538, Hobart, Tasmania 7001 Australia

**Keywords:** Biodiversity, Animal migration, Biogeography

## Abstract

Shedding light on the distribution and ecosystem function of mesopelagic communities in the twilight zone (~200–1000 m depth) of global oceans can bridge the gap in estimates of species biomass, trophic linkages, and carbon sequestration role. Ocean basin-scale bioacoustic data from ships of opportunity programs are increasingly improving this situation by providing spatio-temporal calibrated acoustic snapshots of mesopelagic communities that can mutually complement established global ecosystem, carbon, and biogeochemical models. This data descriptor provides an overview of such bioacoustic data from Australia’s Integrated Marine Observing System (IMOS) Ships of Opportunity (SOOP) Bioacoustics sub-Facility. Until 30 September 2020, more than 600,000 km of data from 22 platforms were processed and made available to a publicly accessible Australian Ocean Data Network (AODN) Portal. Approximately 67% of total data holdings were collected by 13 commercial fishing vessels, fostering collaborations between researchers and ocean industry. IMOS Bioacoustics sub-Facility offers the prospect of acquiring new data, improved insights, and delving into new research challenges for investigating status and trend of mesopelagic ecosystems.

## Abstract (summary)

**Table:** 

Measurement(s)	bioacoustic data • sound quality
Technology Type(s)	echosounder • data processing software
Sample Characteristic - Organism	macro-zooplankton • micronekton
Sample Characteristic - Environment	ocean
Sample Characteristic - Location	Tasman Sea • Southern Ocean • Indian Ocean • South Pacific Ocean • North Pacific Ocean • Coral Sea • North Atlantic Ocean

Machine-accessible metadata file describing the reported data: 10.6084/m9.figshare.13172516

## Background & Summary

**Fig. 1:**
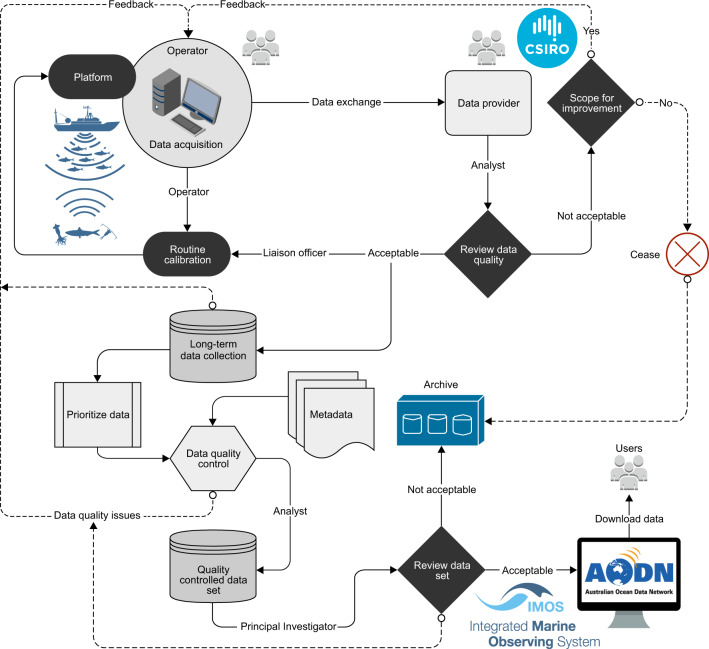
Schematic overview of IMOS Bioacoustics sub-Facility operations to collect and publish bioacoustic data with related metadata. Bioacoustic data received from diverse operators are quality-controlled and made available through the Australian Ocean Data Network (AODN) Portal. IMOS currently has a portfolio of 13 Facilities that undertake systematic and sustained observations of Australia’s marine environment. It is integrated in terms of its geographic domain (from coast to open ocean) and scientific domain, combining a wide range of physical, chemical, and biological observations from a variety of platforms. IMOS observations are turned into data that can be discovered, accessed, downloaded, used, and reused through the data Facility AODN.

**Fig. 2:**
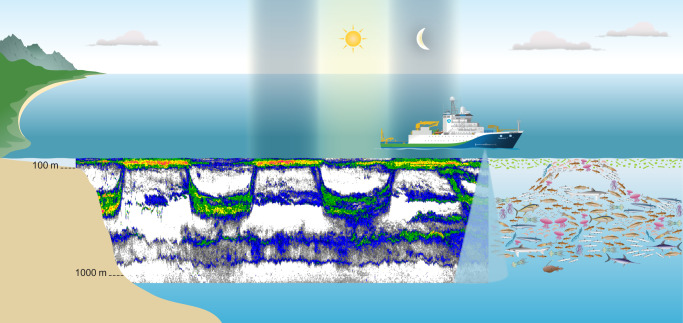
Example of how bioacoustics data is collected from a vessel by transmitting pulses of sound in water that reflects off the organisms to produce an echogram (38 kHz).

Since 2010, as a part of existing ocean industry collaboration, Australia’s Integrated Marine Observing System (IMOS) Ships of Opportunity (SOOP) Bioacoustics sub-Facility (here onwards IMOS Bioacoustics sub-Facility) has been collecting opportunistic, supervised, and unsupervised active bioacoustic data from different platforms including commercial fishing and research vessels transiting ocean basins^1^ (Fig. 1). The resulting acoustic snapshots^2^ provide a proxy for the combined effects of size, abundance, distribution, diversity, and behavior of mid-trophic mesopelagic communities including macro-zooplankton and micronekton in the twilight zone of global oceans (Fig. 2). The broad goal of IMOS Bioacoustics sub-Facility is to provide repeated active bioacoustic observations for the status and trend of ocean life to 1000 m at basin and decadal scales.

The primary data-type derived from IMOS Bioacoustics sub-Facility is the georeferenced, calibrated^3,4^, and processed^5^ single-beam water column volume backscattering coefficient *s*_*v*_ (m^2^ m^−3^) values, representing the linear sum of backscatter from acoustically detectable individual organisms within the sampling volume^2^ (Fig. 2). In suitable circumstances, it is proportional to the density of dominant scattering organisms, and the primary data for estimating biomass from acoustics at regional and global scales using existing^6,7^ and future methods.

Mesopelagic communities are mid-water predators and prey in the twilight zone, and presumed to make the largest natural daily animal movement on earth based on their biomass, revealing diel vertical migration^8,9^ and large-scale spatio-temporal patterns in pelagic sound scattering layers^10^ (Fig. 2). They have been identified as one of the least investigated components of open ocean ecosystems^11^, transferring energy from primary producers to higher predators, and regulating carbon transfer from surface to deep-ocean by linking epipelagic and deep-water food chains^12–16^.

Ships of opportunity bioacoustic sampling methods are useful for cost-effective mapping and biomass estimation of mesopelagic communities at regional and global scales with recognized potentials and challenges^1^. Acoustic estimation of biomass using vessel-based echosounder is complicated by many confounding factors^17^ including lack of accurate taxonomic information about insonified organisms, complex size distribution of scattering organisms, unknown species composition, and frequency-specific selectivity of echosounder measurements^18,19^. From an integrated ocean observing system perspective, a way forward to address these challenges would be to acquire multi-frequency data^20,21^ for improved segregation of dominant scattering groups. With advancing and diverse applications of multi-frequency systems, acoustic methods have been used to classify dominant organisms into gas-filled or fluid-filled categories^22–29^. Such methodologies are improving with the availability of broadband and wideband echosounders^30^ that would help to segment and attribute basin-scale bioacoustic observations into different scattering (or functional) groups.

Despite uncertainties with echosounder calibration, methodological challenges, species identification, and frequency-specific scattering of individual organisms, ships of opportunity bioacoustic sampling methods offer great potential to better understand the structure and ecosystem function of global mesopelagic communities, necessitating continued data acquisition and processing efforts^5,31,32^ with increased global accessibility^33^. The acoustic snapshots revealing spatio-temporal sound scattering patterns, deep scattering layer, and diel vertical migration (Fig. 2) can offer improved ecological insights^34,35^ for marine ecosystem acoustics^36–38^, in addition to established linkages with oceanographic processes^39–48^ and environmental covariates^49–51^ including light^52–54^, oxygen concentration^55–57^, temperature^58,59^, chlorophyll *a*^60^, and primary production^6,59^.

**Fig. 3:**
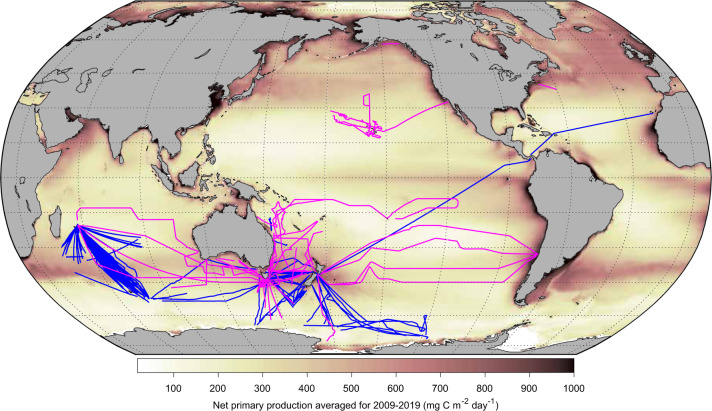
Map showing spatial coverage of bioacoustic data processed until 30 September 2020. Fishing and research vessels are categorized as blue and magenta respectively. A satellite-derived map of ocean net primary production (NPP) averaged for the years 2009–2019 is superimposed. Readers are directed to check the AODN Portal for the latest data set.

**Fig. 4:**
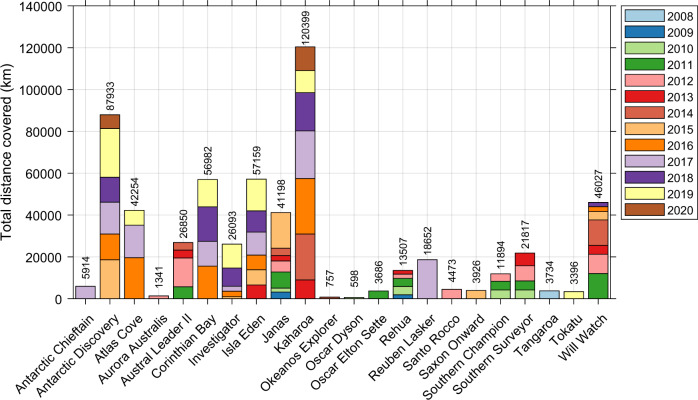
Platforms contributed data to IMOS Bioacoustics sub-Facility in terms of total line kilometres covered by each year.

Currently, 22 platforms have contributed data to IMOS Bioacoustics sub-Facility, including both commercial fishing and research vessels (Table 1). Key time series data sets have been collected across the Tasman Sea, Southern Ocean, and Southern Indian Ocean. The extent of processed bioacoustic data archived under this sub-Facility is expanding with an improved spatial (Fig. 3) and temporal coverage (Fig. 4, until 30 September 2020). The majority of archived data are single-frequency 38 kHz (565,661 km) echosounder observations, but also include growing coverage of multi-frequency 18 kHz (118,260 km), 70 kHz (44,368 km), and 120 kHz (70,400 km) data, highlighting different scattering layers and functional groups.

**Table 1:** List of platforms that contributed data to the IMOS Bioacoustics sub-Facility.

Platform name	Type	Operating frequency (kHz)	Echosounder type	Operator	Area of operation
Antarctic Chieftain	Ship, fishing	18, 38	ES70	Australian Longline Pty Ltd	Southern Indian Ocean
Antarctic Discovery	Ship, fishing	18, 38	ES70/ES80	Australian Longline Pty Ltd	Southern Ocean, Tasman Sea
Atlas Cove	Ship, fishing	18, 38	ES70	Austral Fisheries	Southern Indian Ocean
Aurora Australis	Ship, research	12, 38, 120, 200	EK60	Australian Antarctic Division	Southern Ocean
Austral Leader II	Ship, fishing	38	ES60/ES70	Austral Fisheries	Southern Indian Ocean
Corinthian Bay	Ship, fishing	38	ES70	Austral Fisheries	Southern Indian Ocean
Investigator	Ship, research	18, 38, 70, 120, 200, 333	EK60/EK80	Commonwealth Scientific and Industrial Research Organisation (CSIRO)	Australian Exclusive Economic Zone (EEZ), Tasman Sea, Coral Sea, Southern Ocean
Isla Eden	Ship, fishing	38	ES70	Austral Fisheries	Southern Indian Ocean
Janas	Ship, fishing	38	ES60	Talley’s Group Limited	Southern Indian Ocean, Southern Ocean, Tasman Sea
Kaharoa	Ship, research	38	ES60	National Institute of Water and Atmospheric Research (NIWA)	Southern Indian Ocean, South Pacific Ocean, Tasman Sea
Okeanos Explorer^89^	Ship, research	18, 38, 70, 120, 200	EK60/EK80	National Oceanic and Atmospheric Administration (NOAA)	North Atlantic Ocean, North Pacific Ocean
Oscar Dyson^90^	Ship, research	18, 38, 70, 120, 200	EK60	National Oceanic and Atmospheric Administration (NOAA)	Bering Sea, Gulf of Alaska
Oscar Elton Sette^91^	Ship, research	38, 70, 120, 200	EK60	National Oceanic and Atmospheric Administration (NOAA)	North Pacific Ocean
Rehua	Ship, fishing	38	ES60/ES80	Sealord Group Ltd	Tasman Sea
Reuben Lasker^92^	Ship, research	18, 38, 70, 120, 200, 333	EK60/EK80	National Oceanic and Atmospheric Administration (NOAA)	North Pacific Ocean
Santo Rocco	Ship, fishing	38	ES60	Australian Wild Tuna Pty Ltd	Eastern Australian EEZ
Saxon Onward	Ship, fishing	38	ES60	Voyager Seafoods Pty Ltd	Eastern Australian EEZ, Tasman Sea
Southern Champion	Ship, fishing	38	ES60/ES70	Austral Fisheries	Southern Indian Ocean
Southern Surveyor	Ship, research	38, 120	EK60	Commonwealth Scientific and Industrial Research Organisation (CSIRO)	Australian EEZ, Tasman Sea, Coral Sea, Southern Ocean
Tangaroa	Ship, research	18, 38, 70, 120, 200	EK60	National Institute of Water and Atmospheric Research (NIWA)	Southern Ocean, South Pacific Ocean
Tokatu	Ship, fishing	38, 70	ES80	Sealord Group Ltd	Tasman Sea
Will Watch	Ship, fishing	38	ES70	Sealord Group Ltd	Southern Indian Ocean

Note that operating frequencies 12, 200, and 333 kHz are currently not prioritized and processed due to either calibration issues or range limitations.

The main goals and potential uptake values of IMOS Bioacoustics sub-Facility data are: (1) provide calibrated time series acoustic observations for the status and trend of mesopelagic ecosystem, (2) develop a framework synergizing active bioacoustic observations and ecosystem models^61,62^ for studying open ocean ecosystem dynamics, and (3) develop an active bioacoustic data-based ecosystem Essential Ocean Variable (eEOV)^63^ to complement established and future ocean observing systems measuring physical, chemical, and biological environment of the ocean. These frameworks would help to advance scientific knowledge of marine food chains and manage marine ecosystems sustainably.

## Methods

The terminology used in this data descriptor follows Demer*, et al*.^3^, based mostly on Maclennan*, et al*.^64^. All symbols signifying variables are italicized. Any symbol for a variable (*x*) that is not logarithmically transformed is in lower case. Any symbol for a logarithmically transformed variable, e.g. $X=1{0\log }_{10}\left(x/{x}_{ref}\right)$, with units of decibels referred to *x*_*ref*_  (dB re *x*_*ref*_) is capitalized.

### Echosounder data

In a widely used Simrad echosounder (Table 1), the proprietary format raw data (.raw) from each transmission and reception cycle (here onwards ping) includes received echo power *p*_*er*_ (W), with the General Purpose Transceiver (GPT) settings: frequency *f* (kHz), transmit power *p*_*et*_ (W), pulse duration $\tau$ (s), transducer on-axis gain *G*_0_ (dB re 1), area backscattering coefficient *s*_*a*_ (m^2^ m^–2^) correction factor *S*_*a*_ _corr_ (dB re 1), and equivalent two-way beam angle Ψ (dB re 1 sr) of the transducer. These data and associated settings were used to calculate and display volume backscattering strength ${S}_{v}$ (dB re 1 m^2^ m^−3^) for one or more frequency channels as^3^:

1 $${S}_{v}[i,j]={P}_{er}[i,j]+20\,{\log }_{10}r[i,j]+2{\alpha }_{a}r[i,j]-10\,{\log }_{10}\left(\frac{({p}_{et}{\lambda }^{2}{g}_{0}^{2}{c}_{w}\tau \psi )}{32{\pi }^{2}}\right)-2{S}_{a{\rm{c}}{\rm{o}}{\rm{r}}{\rm{r}}},$$

where ${P}_{er}$ (dB re 1 W) is the received power, $r$ (m) is the range to the target, ${\alpha }_{a}$ (dB m^–1^) is the absorption coefficient, $\lambda$ (m) is the wavelength, ${g}_{0}$ (dimensionless) is the transducer on-axis gain, ${c}_{w}$ (m s^–1^) is the sound speed in water, $\psi$ (sr) is the equivalent two-way beam angle, and the index *i* and *j* represent vertical sample number and horizontal ping number respectively.

### Echosounder calibration

Echosounder calibration is a prerequisite for quantitative bioacoustic studies. The overall on-axis performance of echosounders installed on the participating platforms was routinely evaluated by established sphere calibration method^3,4^. This method provides calibrated ${G}_{0}$ and ${S}_{a{\rm{c}}{\rm{o}}{\rm{r}}{\rm{r}}}$ required for standardizing ${S}_{v}$ data (Eq. 1) collected by diverse platforms with a traceable calibration history. The sphere calibration also provides a check for transducer beam-pattern characteristics and related Ψ. The manufacturer-specified Ψ adjusting for the local sound speed variation at the calibration location was used due to the difficulty in obtaining an independent measurement of hull-mounted transducer beam pattern.

The raw data acquired using ES60 and ES70 echosounders were modulated with a triangle wave error sequence^65^. The triangle wave error (with a 1 dB peak-to-peak amplitude and a 2720 ping period) was removed from calibration data before calculating ${G}_{0}$ and ${S}_{a{\rm{c}}{\rm{o}}{\rm{r}}{\rm{r}}}$. Open ocean transit (here onwards transect) data were not corrected for the triangle wave error due to data management and storage constraints at the start of the program. Generally, this error will average to zero over a full period of 2720 pings for normal operations and 1 km horizontal resolution of the processed data. To facilitate the processing of high-resolution data (e.g. 100 m horizontal resolution) and slow ping rate systems, transect data files were corrected for this error (if applicable) with associated metadata, since September 2020.

### Data acquisition

Ensuring the operational need of participating platforms (e.g. fishing), the data acquisition settings in Table 2 were used to optimize quality and practical utility of collected data. The transmit power was selected based on the recommended^20^ settings for commonly used Simrad echosounders. The pulse duration was chosen as a trade-off between sample resolution and acceptable signal-to-noise ratio (SNR, dB re 1) in the mesopelagic zone, and the logging range was set to provide robust estimates of echosounder background noise (dB re 1 W) levels^66^.

**Table 2:** Commonly used data acquisition settings for IMOS Bioacoustics sub-Facility platforms.

Frequency (kHz)	Transmit power (W)	Pulse duration (ms)	Ping rate	Logging range (m)
18	2000	2.048	Maximum	0–1800
38	2000	2.048	Maximum	0–1800
70	700	2.048	Maximum	0–1800
120	250	1.024	Maximum	0–1800

Note that high-frequency 70 and 120 kHz echosounders are not capable of recording high-quality biological scattering down to 1800 m range. This logging range was set to provide robust estimates of echosounder background noise levels with a presumption that at far ranges the noise will be dominating over the biological scattering due to beam spreading and absorption losses. The absorption of sound in water increases rapidly with frequency and high-frequency echosounders are limited to short ranges.

### Data registration and management

**Fig. 5:**
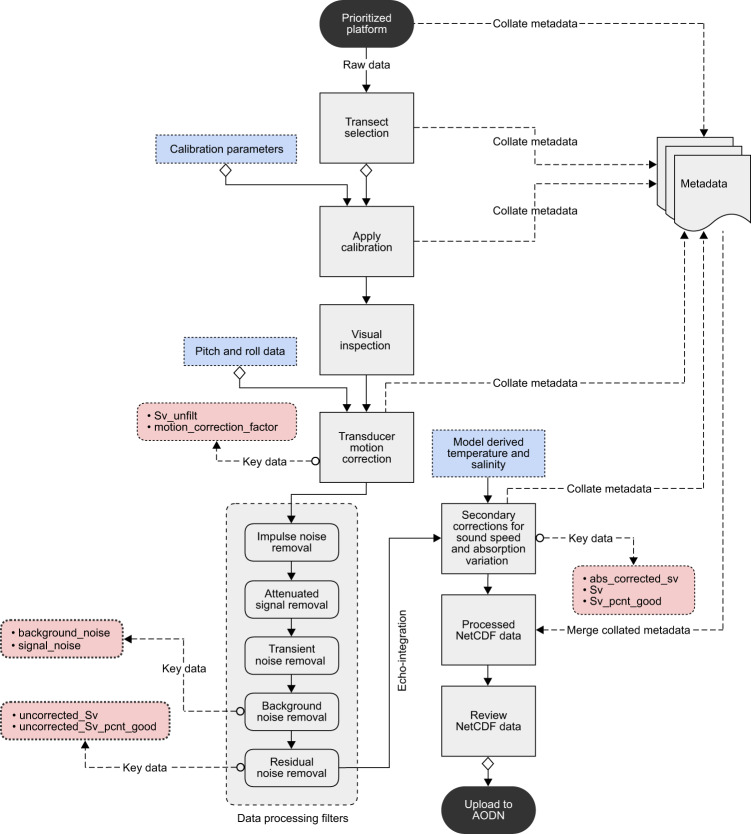
Flowchart of methods implemented to produce quality-controlled bioacoustic data, providing an overview of data processing sequences in the context of key data variables present in a NetCDF file. Note that before transducer motion correction and filtering steps, calibrated ${S}_{v}$ values within each ping were resampled (by taking mean in the linear domain) to a specified vertical resolution of 2 m to smooth out vertical sample-to-sample variations in ${S}_{v}$.

Depending on the primary purpose of participating platforms, raw data received from operators (Table 1) may cover transects and periods of fishing or scientific activities. A custom *Java* software suite was developed to assist data management and help identify transects for post processing (Fig. 5). These tools were used to create information (*inf*) files. The *inf* file is in plain text format that contains user-defined metadata (platform name, relevant platform call sign, voyage name, transect attributes, and relevant comments). It also includes key data acquisition settings extracted from the raw data files including frequency, transmit power, pulse duration, and echosounder details (GPT channel identifier and transducer model). The platform navigation details (total travel time, total distance covered, and average platform speed), temporal extent (start and end time of data volume), and geographic extent (limits of latitude and longitude) were also captured in the *inf* files. These *inf* files were checked for consistent data acquisition settings, transect selection, and excluding continental shelf water column backscatter data. Raw data files with inconsistent data acquisition or unknown calibration settings were not considered for further processing and archived locally.

### Data processing routines

Data sets were initially processed using Echoview® software (Echoview Software Pty Ltd, Hobart, Tasmania, Australia) that includes a sequence of data processing filters^5^ designed to remove noise and improve data quality. Transect data files applying related time offset to Coordinated Universal Time (UTC) and calibration parameters were visualized (Eq. 1) as frequency-specific echograms in Echoview® for visual inspection, transducer motion correction, and filtering processes (Fig. 5). Subsequent processing and packaging were completed using MATLAB® software (MathWorks, Natick, Massachusetts, USA). All processing steps were semi-automated using a custom MATLAB® Graphical User Interface (GUI) integrated with Component Object Model (COM) objects controlling Echoview® software.

### Visual inspection of data

Acoustic data quality from different platforms can vary significantly due to signal attenuation (i.e. attenuation of transmit and/or received signal to a level below the analysis threshold), and signal degradation due to combined transducer motion and noise. Data quality control involved visual inspection of echograms (Fig. 5), followed by marking the seabed (if present) and regions of bad data using echogram tools available in Echoview®. Pings with prolonged noise interference or signal attenuation were flagged as bad data. Data shallower than 10 m were removed to exclude echosounder transmit pulse and echoes in the transducer nearfield. Similarly, data deeper than the seabed (if present) were removed from the analyses. Additionally, regions of aliased seabed echoes (i.e. seabed reverberations from preceding pings coinciding with the current ping) were manually flagged as bad data. Valid high scattering from biological sources (e.g. pelagic fish schools that may occur between surface and 250 m depth) causing an apparent transition in backscatter intensities was manually preserved from the transient noise filter described below^5^.

### Transducer motion correction

Echo-integration results will be biased if the change in orientation of transducer beam between the times of each ping is not accounted for. The effect of transducer motion on echo-integration was studied by Stanton^67^ and later Dunford^68^ developed a single correction function that can be applied for a wide range of circular transducers and related ${s}_{v}$ data. To fully characterize platform movement, the Dunford^68^ algorithm implemented in Echoview® requires motion data (i.e. pitch and roll of a platform) recorded at a rate above the Nyquist rate of platform’s angular motion^69^ to avoid temporal aliasing due to an inadequate sampling rate. When platform motion data were available at a suitable sampling rate (see ‘Technical Validation’ section), transducer motion effects were corrected using Dunford^68^ algorithm by ensuring time synchronization with recorded acoustic data (Fig. 5).

### Data processing filters

Fishing vessels (FV) contributing to IMOS Bioacoustics sub-Facility were not purposely built for collecting high-quality bioacoustic data. Various factors including inclement weather and vessel design can affect data quality that could cause large biases in derived ${s}_{v}$ values. To minimize these biases, data processing filters were applied to the raw data (Fig. 5). Transducer motion-corrected data were subject to a sequence of data processing filters^5^ designed to mitigate impulse noise, signal attenuation, transient noise, and background noise^66^.

Data processing filters were applied to each ${S}_{v}$ sample in an echogram, identified by a vertical sample number $i$ and horizontal ping number $j$. The ‘context window’ defined for filters include a current ping, and surrounding pings on either side of the current ping. Depending on the filter used, the context window either centres on the current ping or current sample, and slides over the entire echogram.

### Impulse noise removal

Impulse noise affects discrete sections of the data with a duration of less than one ping, for example, transmit pulses originated from other unsynchronized acoustic systems. The impulse noise removal algorithm implemented in Echoview® (based on Ryan*, et al*.^5^) compares each ${S}_{v}$ sample in a current ping to the adjacent ${S}_{v}$ samples (at the same depth) in surrounding pings defined by a context window of specified width $W$ (see details of context window in Table 3). A smoothed copy of original ${S}_{v}$ values (i.e. unfiltered data) within the context window was used to identify impulse noise (see details of smoothing window in Table 3). The original ${S}_{v}$ samples were identified as impulse noise if the corresponding smoothed ${S}_{v}$ samples satisfy the condition:

2 $${S}_{v}[i,j]-{S}_{v}[i,j-m] > \delta \,{\rm{a}}{\rm{n}}{\rm{d}}\,{S}_{v}[i,j]-{S}_{v}[i,j+n] > \delta ,$$

where ${S}_{v}[i,j]$ (dB re 1 m^2^ m^−3^) represents smoothed copy of current ping with a vertical sample number $i$ and horizontal ping number $j$, $m$ and $n$ are the positive integer offsets from the current ping determined by the width ($W$) of context window, where $m,n\in \left\{1,\ldots ,\frac{W-1}{2}\right\}$ and $W$ is an odd integer value in the range 3 to 9, and $\delta$ (dB re 1 m^2^ m^−3^) is an empirically determined impulse noise removal threshold value. Identified noise values were replaced as ‘no data’. The impulse noise removal parameters defined in Echoview® are given in Table 3.

**Table 3:** User-defined impulse noise removal parameters in Echoview®.

Filter parameter	Unit	Description	Value used
Exclusion threshold	dB re 1 m^2^ m^−3^	The value of a time-varied threshold *TVT*(*r*) (dB re 1 m^2^ m^−3^) defined at 1 m range. This threshold will vary as a function of range from the transducer as: $TVT(r)={S}_{v}\left(1\right)+20\,{{\rm{\log }}}_{10}r+2{\alpha }_{a}(r-1)$, where ${S}_{v}\left(1\right)$ (dB re 1 m^2^ m^−3^) is the volume backscattering strength at one-meter range *r* (m) and ${\alpha }_{a}$ (dB m^–1^) is the absorption coefficient. Any ${S}_{v}(r)$ values below this calculated $TVT(r)$ were preserved from the impulse noise filter.	−170
Vertical size of smoothing window	Metre	Vertical window size used for smoothing. Corresponding horizontal smoothing window is one ping wide.	5
Horizontal size of context window ($W$)	Number	Width of the context window (i.e. number of pings including the current ping) used to identify noise.	3
Detection threshold ($\delta$)	dB re 1 m^2^ m^−3^	The impulse noise removal threshold value.	6

### Attenuated signal removal

Signal attenuation is generally caused by air bubbles beneath the transducer that may occur for one ping or can persist over multiple pings. The attenuated signal removal algorithm implemented in Echoview® (based on Ryan*, et al*.^5^) compares the percentile score of ${S}_{v}$ samples in a current ping with the percentile score of ${S}_{v}$ samples in surrounding pings defined by a context window (see details of context window in Table 4). The current ping was removed and replaced as ‘no data’ if:

3 $$p({S}_{v}[m\times n])-p({S}_{v}[i,j])\ge \delta ,$$

where the symbol $p$ denotes the desired percentile value, ${S}_{v}[i,j]$ (dB re 1 m^2^ m^−3^) is the current ping with a vertical sample number $i$ and horizontal ping number $j$, ${S}_{v}\left[m\times n\right]$ (dB re 1 m^2^ m^−3^) represents ${S}_{v}$ samples in the context window defined by $m$ vertical samples and $n$ horizontal pings, and $\delta$ (dB re 1 m^2^ m^−3^) is an empirically determined attenuated signal removal threshold value. The attenuated signal removal parameters defined in Echoview® are given in Table 4.

**Table 4:** User-defined attenuated signal removal parameters in Echoview®.

Filter parameter	Unit	Description	Value used
Exclude above depth	Metre	Nominal upper limit of deep scattering layer (DSL) depth. ${S}_{v}$ samples between surface and this depth were not included in the algorithm (i.e. Eq. 3). Note that this depth line (in synchronous with ‘exclude below depth’ line) was adjusted to track high signal homogeneous regions for all frequencies. Ryan*, et al*.^5^ observed robust performance of attenuated signal algorithm in the DSL where SNR and ping overlap is generally high.	500
Exclude below depth	Metre	Nominal lower limit of DSL depth. ${S}_{v}$ samples below this depth were not included in the algorithm (i.e. Eq. 3). Note that this depth line (in synchronous with ‘exclude above depth’ line) was adjusted to track high signal homogeneous regions for all frequencies.	600
Vertical size of context window ($m$)	Metre	Vertical size of the context window used to identify pings with attenuated signal. This window size defines the vertical separation between ‘exclude above’ and ‘exclude below’ depth lines (see above).	100
Horizontal size of context window ($n$)	Number	Horizontal size of the context window (i.e. number of pings) used to identify pings with attenuated signal.	301
Detection percentile ($p$)	Percentile	The percentile value used for comparison between the current ping and context window.	50
Detection threshold ($\delta$)	dB re 1 m^2^ m^−3^	The threshold value used to identify pings with attenuated signal.	8

### Transient noise removal

Transient noise is introduced to the received signal that can occur at irregular intervals and persists over multiple pings. The transient noise removal algorithm implemented in Echoview® (based on Ryan*, et al*.^5^) compares each ${S}_{v}$ sample in a current ping with the percentile score of ${S}_{v}$ samples in surrounding pings defined by a context window (see details of context window in Table 5). A smoothed copy of original ${S}_{v}$ values (i.e. unfiltered data) within the context window was used to identify noise (see details of smoothing window in Table 5). The original ${S}_{v}$ samples were identified as transient noise if the corresponding smoothed ${S}_{v}$ samples satisfy the condition:

4 $${S}_{v}[i,j]-p\left({S}_{v}\left[m\times n\right]\right) > \delta ,$$

where the symbol $p$ denotes the desired percentile value, ${S}_{v}[i,j]$ (dB re 1 m^2^ m^−3^) represents smoothed copy of current ping with a vertical sample number $i$ and horizontal ping number $j$, ${S}_{v}\left[m\times n\right]$ (dB re 1 m^2^ m^−3^) represents smoothed copy of ${S}_{v}$ samples in the context window defined by $m$ vertical samples and $n$ horizontal pings, and $\delta$ (dB re 1 m^2^ m^−3^) is an empirically determined transient noise removal threshold value. Identified noise values were replaced as ‘no data’. The transient noise removal parameters defined in Echoview® are given in Table 5.

**Table 5:** User-defined transient noise removal parameters in Echoview®.

Filter parameter	Unit	Description	Value used
Exclude above depth	Metre	Nominal depth line. Filter is not applied between surface and this depth. Note that this depth line has been adjusted to preserve valid high scattering from biological sources.	250
Exclusion threshold	dB re 1 m^2^ m^−3^	The value of a time-varied threshold $TVT(r)$ defined at 1 m range. See Table 3 for more details. Any ${S}_{v}(r)$ values below this calculated $TVT(r)$ were preserved from the transient noise filter.	−150
Vertical size of smoothing window	Metre	Vertical window size used for smoothing. Corresponding horizontal smoothing window is one ping wide.	20
Vertical size of context window ($m$)	Number	Vertical size of the context window (i.e. number of samples) used to identify noise.	11
Horizontal size of context window ($n$)	Number	Horizontal size of the context window (i.e. number of pings) used to identify noise.	51
Detection percentile ($p$)	Percentile	The value used to calculate percentile of ${S}_{v}$ samples in the context window.	15
Detection threshold ($\delta$)	dB re 1 m^2^ m^−3^	The transient noise removal threshold value.	15

### Background noise removal

Background noise is introduced to the received signal that can vary in intensity and pattern (see section ‘Technical Validation’). According to De Robertis and Higginbottom^66^, the calibrated ${S}_{v}$ values (Eq. 1) can be expressed as the sum of contributions from the signal and noise as:

5 $${S}_{{v}_{{\rm{cal}}}}[i,j]=10\,{{\rm{\log }}}_{10}\left(1{0}^{\left({S}_{{v}_{{\rm{signal}}}}[i,j]/10\right)}+1{0}^{\left({S}_{{v}_{{\rm{noise}}}}[i,j]/10\right)}\right),$$

where ${S}_{{v}_{{\rm{cal}}}}$ (dB re 1 m^2^ m^−3^) is the calibrated ${S}_{v}$ samples derived from the raw data (i.e. Eq. 1), ${S}_{{v}_{{\rm{signal}}}}$ (dB re 1 m^2^ m^−3^) is the calibrated ${S}_{v}$ samples representing the contribution from signal, ${S}_{{v}_{{\rm{noise}}}}$ (dB re 1 m^2^ m^−3^) is the calibrated ${S}_{v}$ samples representing the contribution from noise, and the index *i* and *j* represent vertical sample number and horizontal ping number respectively.

To estimate background noise levels, calibrated received power ${P}_{e{r}_{{\rm{cal}}}}[i,j]$ (dB re 1 W) values were calculated from ${S}_{{v}_{{\rm{cal}}}}[i,j]$ values by subtracting the time-varied gain (TVG) function^2^ (i.e. $2{0\log }_{10}r+2{\alpha }_{a}r$) from Eq. 1 as:

6 $${P}_{e{r}_{{\rm{cal}}}}[i,j]={S}_{{v}_{{\rm{cal}}}}[i,j]-20\,{{\rm{\log }}}_{10}r[i,j]-2{\alpha }_{a}r[i,j].$$

The calibrated ${P}_{e{r}_{{\rm{cal}}}}[i,j]$ values were averaged^66^ (in linear domain) within an ‘averaging cell’ of $M$ vertical samples (with an index $k$) and $N$ horizontal pings (with an index $l$) to estimate noise as:

7 $$Noise\left(l\right)={\rm{\min }}(\bar{{P}_{e{r}_{{\rm{cal}}}}}[k,l]),$$

where $\bar{{P}_{e{r}_{{\rm{cal}}}}}[k,l]$ (dB re 1 W) is the averaged ${P}_{e{r}_{{\rm{cal}}}}[i,j]$ values calculated for each averaging cell with a vertical sample interval $k$ and horizontal ping interval $l$, and $Noise\left(l\right)$ (dB re 1 W) is the representative noise estimate for the ‘middle ping’ in each horizontal interval $l$. Note that the averaging cell slides over the entire echogram (see details of averaging cell in Table 6).

**Table 6:** User-defined background noise removal parameters in Echoview®.

Filter parameter	Unit	Description	Value used
Vertical size of averaging cell ($M$)	Number	The vertical size of the averaging cell (i.e. number of samples). This cell height defines the range interval for noise estimation (see Eq. 7).	15
Horizontal size of averaging cell ($N$)	Number	The horizontal size of the averaging cell (i.e. number of pings). This cell width defines the ping interval for noise estimation.	10
Vertical overlap of averaging cell	%	The percentage vertical overlap of the averaging cell.	0
Maximum noise threshold ($Nois{e}_{max}$)	dB re 1 W	The upper limit of background noise levels. Any noise estimates greater than this threshold was replaced with the ‘value used’.	−100
Minimum SNR threshold ($Minimu{m}_{SNR}$)	dB re 1	Acceptable SNR for background noise corrected data. Corrected ${S}_{v}$ data with corresponding SNR values below this threshold were set to ‘−999’. This low SNR threshold was empirically determined to preserve weak scattering signal.	0.1

An empirically determined maximum threshold $Nois{e}_{max}$ (dB re 1 W) (see Table 6) was applied to $Noise\left(l\right)$ values as an upper limit of background noise levels. Any $Noise\left(l\right)$ values exceeding this threshold was replaced with the predefined $Nois{e}_{max}$ value.

The $Noise\left(l\right)$ value estimated for a given horizontal ping interval $l$ was assigned to all individual pings constituting the interval to establish noise $Noise\left(j\right)$ (dB re 1 W) estimate for each ping. The effect of TVG was added to the $Noise\left(j\right)$ levels to compute ${S}_{{v}_{{\rm{noise}}}}$ for each vertical sample number $i$ and horizontal ping number $j$ as:

8 $${S}_{{v}_{{\rm{noise}}}}[i,j]=Noise\left(j\right)+20\,{{\rm{\log }}}_{10}r\left[i,j\right]+2{\alpha }_{a}r[i,j].$$

The background noise corrected volume backscattering strength ${S}_{{v}_{{\rm{bnc}}}}[i,j]$ (dB re 1 m^2^ m^−3^) values for each vertical sample number $i$ and horizontal ping number $j$ were estimated as:

9 $${S}_{{v}_{{\rm{bnc}}}}[i,j]=10\,{{\rm{\log }}}_{10}\left(1{0}^{\left({S}_{{v}_{{\rm{cal}}}}[i,j]/10\right)}-1{0}^{\left({S}_{{v}_{{\rm{noise}}}}[i,j]/10\right)}\right).$$

The SNR, a measure of the relative contribution of signal and noise was estimated as:

10 $$SNR[i,j]={S}_{{v}_{{\rm{bnc}}}}[i,j]-{S}_{{v}_{{\rm{noise}}}}[i,j],$$

where $SNR[i,j]$ (dB re 1) is the signal-to-noise ratio for each vertical sample number $i$ and horizontal ping number $j$.

An empirically determined threshold $Minimu{m}_{SNR}$ (dB re 1) (see Table 6) was used as an acceptable SNR for background noise corrected ${S}_{{v}_{{\rm{bnc}}}}[i,j]$ data. The ${S}_{{v}_{{\rm{bnc}}}}[i,j]$ values with corresponding $SNR[i,j]$ below this threshold were set to ‘−999’ dB re 1 m^2^ m^−3^ (an approximation of zero in the linear domain). The background noise removal parameters defined in Echoview® are given in Table 6.

### Residual noise removal

In the final stage, a 7 × 7 median filter was applied to remove residual noise retained in the core filtering stages (especially at far ranges). A median filter replaces the current ${S}_{v}$ sample with the median value of ${S}_{v}$ samples in a $M$ × $M$ neighbourhood. It is important to note that the output of 7 × 7 median filter was not directly used for echo-integration, rather it was used to flag residual noise retained from the core filtering process. A maximum data threshold of −50 dB re 1 m^2^ m^−3^ and a time-varied threshold $TVT(r)$ with the reference value of −160 dB re 1 m^2^ m^−3^ (defined at 1 m range) was applied to the background noise corrected ${S}_{{v}_{{\rm{bnc}}}}[i,j]$ data before applying 7 × 7 median filter (see Table 3 for a description of time-varied threshold). ${S}_{{v}_{{\rm{bnc}}}}[i,j]$ values above the maximum threshold (i.e. −50 dB re 1 m^2^ m^-3^) were set to ‘−999’ dB re 1 m^2^ m^−3^. Similarly, ${S}_{{v}_{{\rm{bnc}}}}[i,j]$ values below the calculated $TVT(r)$ values were set to ‘−999’ dB re 1 m^2^ m^-3^ (note that median filter may replace ‘−999’ with the median of samples in the 7 × 7 neighbourhood). The output of the median filter was used to create a Boolean data range bitmap (between −998 to −20 dB re 1 m^2^ m^−3^) with ‘true’ or ‘false’ values for each sample. This Boolean data range bitmap was applied to the background noise corrected ${S}_{{v}_{{\rm{bnc}}}}[i,j]$ data for removing any residual noise before echo-integration. ${S}_{{v}_{{\rm{bnc}}}}[i,j]$ values corresponding to ‘false’ values in the data range bitmap were set to ‘−999’ dB re 1 m^2^ m^−3^.

Quality-controlled ${S}_{v}$ data along with: (1) calibrated and motion corrected raw data, (2) transducer motion correction factor (i.e. difference between ‘motion corrected’ and ‘calibrated raw’ data), (3) background noise, and (4) SNR were exported from Echoview® as echo-integration cells (i.e. grid on an echogram) with a resolution of 1 km horizontal distance (i.e. ping-axis interval $p$) and 10 m vertical depth (i.e. range-axis interval $r$). Echo-integration values were stored as comma-separated values (CSV) files. Exported ${S}_{v}$ data were converted to linear scale for further processing and packaging in MATLAB® (Fig. 5).

### Secondary corrections for sound speed and absorption variation

Quality-controlled ${S}_{v}$ data were echo-integrated and exported using a nominal sound speed ${c}_{w}$ (m s^−1^) and absorption coefficient ${\alpha }_{a}$ (dB m^−1^) values estimated using the equations of Mackenzie^70^ and Francois and Garrison^71^ respectively (see sound speed and absorption coefficient variables in Eq. 1 used for ${S}_{v}$ calculation). However, open ocean transects pass through different hydrographical conditions, so a secondary range dependent correction was required to account for the changes in horizontal and vertical cumulative mean sound speed and absorption as:

11 $$\bar{{S}_{{v}_{{\rm{corr}}}}}[r,p]=\bar{{S}_{{v}_{{\rm{uncorr}}}}}[r,p]+20\,{{\rm{\log }}}_{10}\left(\frac{\bar{{c}_{w}}\left[r,p\right]}{{c}_{w}}\right)+2{r}_{n}[r,p]\left(\bar{{\alpha }_{a}}[r,p]\frac{\bar{{c}_{w}}\left[r,p\right]}{{c}_{w}}\,-\,{\alpha }_{a}\right)-10\,{{\rm{\log }}}_{10}\left(\frac{{c}_{w}[r,p]}{{c}_{w}}\right),$$

or in linear terms:

12 $$\bar{{s}_{{v}_{{\rm{corr}}}}}[r,p]=\frac{\bar{{s}_{{v}_{{\rm{uncorr}}}}}[r,p]{\left(\frac{\bar{{c}_{w}}[r,p]}{{c}_{w}}\right)}^{2}1{0}^{\frac{2{r}_{n}[r,p]}{10}\left(\bar{{\alpha }_{a}}[r,p]\frac{\bar{{c}_{w}}[r,p]}{{c}_{w}}-{\alpha }_{a}\right)}}{\left(\frac{{c}_{w}[r,p]}{{c}_{w}}\right)},$$

where $\bar{{s}_{{v}_{{\rm{uncorr}}}}}[r,p]$ (m^2^ m^−3^) is the uncorrected (but filtered) volume backscattering coefficient values exported from Echoview® at the specified range-axis interval $r$ (i.e. 10 m) and ping-axis interval $p$ (i.e. 1 km), ${r}_{n}[r,p]$ (m) is the regularly spaced depth values for each echo-integration cell, $\bar{{c}_{w}}\left[r,p\right]=\frac{{\sum }_{r=1}^{n}{c}_{w}\left[r,p\right]}{n};\,\forall p$ (m s^−1^) is the cumulative mean sound speed values estimated at each echo-integration cell for the new range ${r}_{a}[r,p]={r}_{n}[r,p]\frac{\bar{{c}_{w}}[r,p]}{{c}_{w}}$ (m) calculation, $\bar{{\alpha }_{a}}\left[r,p\right]=10\,{{\rm{\log }}}_{10}\left(\frac{{\sum }_{r=1}^{n}1{0}^{\left(\frac{{\alpha }_{a}\left[r,p\right]}{10}\right)}}{n}\right);\forall p$ (dB m^−1^) is the cumulative mean absorption coefficient values ‘interpolated’ at the new range ${r}_{a}[r,p]$, and $\bar{{s}_{{v}_{{\rm{corr}}}}}[r,p]$ (m^2^ m^−3^) is the corrected volume backscattering coefficient values at the new range ${r}_{a}[r,p]$.

Due to changes in cumulative mean sound speed, this correction step creates a grid with irregular ${r}_{a}[r,p]$ values. Therefore, the $\bar{{s}_{{v}_{{\rm{corr}}}}}[r,p]$ values at the new ranges ${r}_{a}[r,p]$ were interpolated and reported at the regularly spaced ${r}_{n}[r,p]$ values.

The sound speed and absorption coefficient values for secondary corrections were estimated using the equations of Mackenzie^70^ and Francois and Garrison^71^ respectively. Francois and Garrison^71^ estimate their ‘total absorption equation’ to be accurate within 5% for ocean temperature values of −1.8–30 °C, frequencies of 0.4–1000 kHz, and salinity values of 30–35 PSU. The typical hydrographical conditions (temperature values of 0–27 °C and salinity values of 34–36 PSU) present along the open ocean transects are generally within the reliability limits of Francois and Garrison^71^ equation.

The temperature and salinity data for sound speed and absorption coefficient calculations were interpolated from either CSIRO Atlas of Regional Seas^72^ (CARS, http://www.marine.csiro.au/~dunn/cars2009/ version 2009) or Synthetic Temperature and Salinity (SynTS)^73^ analyses (http://www.marine.csiro.au/eez_data/doc/synTS.html), but can also be derived from oceanographic reanalysis and ocean circulation models. CARS2009 is a digital climatology or atlas of seasonal ocean water properties. It is based on a comprehensive set of quality‐controlled vertical profiles of *in situ* ocean properties (i.e. temperature, salinity, oxygen, nitrate, silicate, and phosphate) collected between 1950 and 2008. CARS2009 NetCDF files contain a gridded mean of these ocean properties and average seasonal cycles generated from the collated observations. CARS2009 covers global oceans on a 0.5 × 0.5 degree grid spatial resolution, and are mapped onto 79 standard depth levels from the sea surface to 5500 m (from this vertical profiles of ocean properties along a bioacoustic transect can be extracted). SynTS is a daily three-dimensional (3D) temperature and salinity product generated by CSIRO, where the CARS temperature and salinity fields are adjusted with daily satellite sea surface temperature (SST) and gridded sea level anomaly (GSLA). SynTS has a 0.2 × 0.2 degree grid spatial resolution, and is mapped onto 66 standard depth levels from the sea surface to 2000 m. Due to limited spatial coverage (60°S–10°N and 90°E–180°E), the SynTS products may not always cover the transect region (e.g. Southern Indian Ocean), in that case CARS climatology values were used for the secondary corrections (Fig. 5).

### Data review, packaging and submission routines

**Fig. 6:**
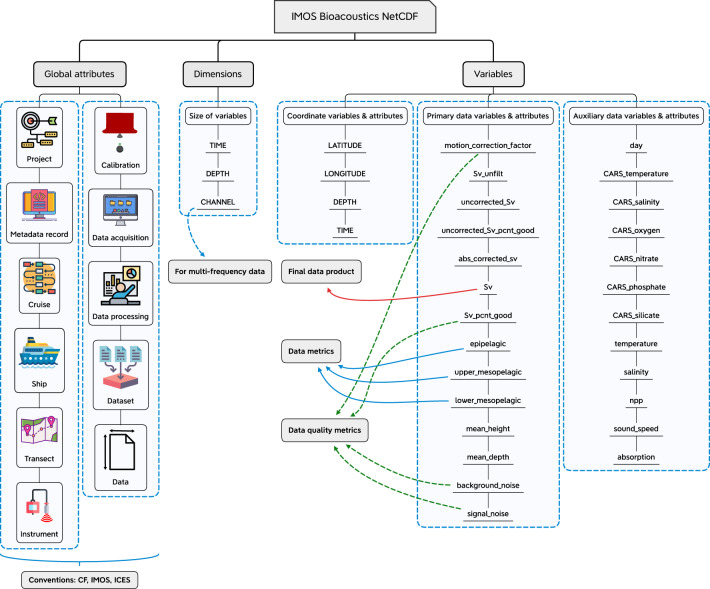
Primary components and organization of key variables present in a NetCDF file with illustrations of key metadata categories. A brief description of these key variables is given in Table 7.

For each processed transect, secondary corrections applied $\bar{{s}_{{v}_{{\rm{corr}}}}}$ data together with metrics of data quality and other auxiliary data variables were stored in Network Common Data Form (NetCDF, www.unidata.ucar.edu) file (NetCDF-4 format) with a resolution of 1 km horizontal distance (i.e. ping-axis interval) and 10 m vertical depth (i.e. range-axis interval) (see ‘Data Records’ section for data contents). This NetCDF file conforms standardized naming conventions and metadata content defined by the Climate and Forecast (CF)^74^, IMOS^75^, and International Council for the Exploration of the Sea (ICES)^76^ published over the years (Fig. 6).

Processed NetCDF files were independently reviewed by both analyst and principal investigator to further investigate data quality. If suitable, the NetCDF file along with ancillary files: (1) acquired raw data (.raw files), (2) platform track in CSV format (containing date, time, latitude, longitude, and time offset to UTC), (3) platform motion data (if recorded) in CSV format (including date, time, pitch, and roll measurements), and (4) a snapshot of processed echogram as Portable Network Graphics (PNG) format were packaged and submitted to the publicly accessible AODN Portal (Fig. 5).

## Data Records

The primary components of a processed NetCDF file are shown in Fig. 6 and described in Table 7 to provide an overview of data contents and structure. Each variable in a NetCDF file is described with an associated description, specifying the data output resulting from each data-collection or analytical step (Table 7).

**Table 7:** Description of key variables present in a NetCDF file.

Components	Description
**Global attributes**	The global attribute section of a NetCDF file contains mandatory metadata that describes general contents and facilitates data discovery. This section is composed of the following key attributes: project, metadata record, cruise, ship, transect, instrument, calibration, data acquisition, data processing, dataset, and data. Note that global attribute names are case sensitive.
**Dimensions**	Dimensions provide information on the size of data variables contained in a NetCDF file, and additionally match coordinate variables to data variables. The dimensions of a data variable define the axes (i.e. TIME and DEPTH) of the quantity it contains.
**Variables**	NetCDF variables include coordinate variables, data and data quality metrics derived from the echosounder primary measurement (i.e. received power), and environmental parameters as given below.
**Coordinate variables**	Coordinate variables locate the data in space and time.
LATITUDE	Specified in decimal degrees relative to the World Geodetic System (WGS84) coordinate reference system.
LONGITUDE	Specified in decimal degrees relative to the WGS84 coordinate reference system.
DEPTH	Measures the depth (m) below the sea surface that is positive in downward direction.
TIME	Represented as decimal number of days since the reference time of 1950-01-01 00:00:00 UTC.
**Primary data variables**	Contains data and data quality metrics derived from the echosounder primary measurement.
motion_correction_factor	Percentage correction applied to calibrated raw ${s}_{v}$ values for transducer motion correction (if platform motion data is available). This variable is the percentage difference between calibrated raw ${s}_{v}$ values before and after applying transducer motion correction algorithm.
Sv_unfilt	Unprocessed mean ${s}_{v}$ values (m^2^ m^−3^). These are an echo-integration of calibrated and transducer motion corrected acoustic data.
uncorrected_Sv	Filtered mean ${s}_{v}$ values (m^2^ m^−3^). These are an echo-integration of calibrated, transducer motion corrected, and filtered acoustic data.
uncorrected_Sv_pcnt_good	Percentage of ${s}_{v}$ data retained after filtering and before secondary corrections.
abs_corrected_sv	Filtered and secondary corrections applied mean ${s}_{v}$ values (m^2^ m^−3^) before depth interpolation.
Sv	Processed mean ${s}_{v}$ values (m^2^ m^−3^). This is the final data product.
Sv_pcnt_good	Percentage of ${s}_{v}$ data retained at the end of post-processing.
epipelagic	Processed ${s}_{v}$ values averaged between 20–200 m depth and converted to decibel (dB re 1 m^2^ m^−3^).
upper_mesopelagic	Processed ${s}_{v}$ values averaged between 200–400 m depth and converted to decibel (dB re 1 m^2^ m^−3^).
lower_mesopelagic	Processed ${s}_{v}$ values averaged between 400–800 m depth and converted to decibel (dB re 1 m^2^ m^−3^).
mean_height	Mean height (m) values for each echo-integration cell. This variable reports the mean height of the echo-integration cell (i.e. grid on an echogram) analyzed. It has been calculated as $T=\frac{{\sum }_{p}^{{A}_{p}}{t}_{p}}{{N}_{p}}$, where $T$ is the mean height (m), ${A}_{p}$ is the set of pings $p$ in the cell, ${N}_{p}$ is the number of pings in the cell, and ${t}_{p}$ is the calculated thickness (m) of the ping $p$. For ${S}_{v}$ echograms, the thickness ${t}_{p}$ is calculated as ${t}_{p}=\Delta {t}_{p}\mathop{\sum }\limits_{s}^{{A}_{s}}{\varepsilon }_{s}$, where ${A}_{s}$ is the set of samples $s$ in the ping $p$, and $\Delta {t}_{p}$ is the thickness (m) of one sample (i.e. the sample spacing for the ping $p$). The symbol ${\varepsilon }_{s}$ is defined as ‘0’ if the sample $s$ is excluded from the analyses or if it is a no-data sample, otherwise ${\varepsilon }_{s}$ is defined as ‘1’.
mean_depth	Mean depth (m) values for each echo-integration cell. This variable reports the mean depth of the echo-integration cell (i.e. grid on an echogram) analyzed. It has been calculated as $\bar{r}=\frac{\mathop{\sum }\limits_{s}^{{A}_{s}}{\varepsilon }_{s}{r}_{s}}{{\sum }_{s}^{{A}_{s}}{\varepsilon }_{s}}$, where *A*_*s*_ is the set of samples *s* in the cell, ${r}_{s}$ is the range of sample *s* in the cell, and $\bar{r}$ is the mean range (m) of samples in the cell. The symbol ${\varepsilon }_{s}$ is defined as ‘0’ if the sample *s* is excluded from the analyses or if it is a no-data sample, otherwise ${\varepsilon }_{s}$ is defined as ‘1’.
background_noise	Background noise (dB re 1 W) values for each ping-axis interval (i.e. horizontal distance). See Eq. 7 for more information.
signal_noise	Signal-to-noise-ratio (dB re 1) for each echo-integration cell. See Eq. 10 for more information.
**Auxiliary data variables**	Auxiliary data variables contain environmental parameters such as climatology and satellite derived data.
day	Diurnal sun cycle information for each ping-axis interval. The numbers 1 (Day), 2 (Sunset ± 1 hr), 3 (Sunrise ± 1 hr), and 4 (Night) are used to represent sun cycle.
CARS_temperature	CARS derived climatology temperature (°C) values for each echo-integration cell.
CARS_salinity	CARS derived climatology salinity (PSU) values for each echo-integration cell.
CARS_oxygen	CARS derived climatology oxygen (ml l^−1^) values for each echo-integration cell.
CARS_nitrate	CARS derived climatology nitrate (µmol l^−1^) values for each echo-integration cell.
CARS_phosphate	CARS derived climatology phosphate (µmol l^−1^) values for each echo-integration cell.
CARS_silicate	CARS derived climatology silicate (µmol l^−1^) values for each echo-integration cell.
temperature	Inferred temperature (°C) values derived from SynTS products for each echo-integration cell. If SynTS is not covering the transect region, CARS_temperature values are substituted to keep consistent data record.
salinity	Inferred salinity (PSU) values derived from SynTS products for each echo-integration cell. If SynTS is not covering the transect region, CARS_salinity values are substituted to keep consistent data record.
npp	Ocean net primary production (NPP, mg C m^−2^ day^−1^) values interpolated for each ping-axis interval (averaged for the previous 12 months with reference to the transect start date). NPP values are based on the Vertically Generalized Production Model (VGPM, http://sites.science.oregonstate.edu/ocean.productivity/standard.product.php).
sound_speed	Sound speed (m s^−1^) in water calculated for each echo-integration cell.
absorption	Absorption coefficient (dB m^−1^) of sound in water calculated for each echo-integration cell.

These variables are described with mandatory variable attributes, linking associated quality flags as ancillary variables (not applicable to all variables in a file). Quality flags provide an assessment of quality control performed.

Processed NetCDF files are published via the Australian Ocean Data Network (AODN) Portal at:

https://portal.aodn.org.au/search?uuid=8edf509b-1481-48fd-b9c5-b95b42247f82.

This portal allows transect selection and data download with spatial and temporal subset options implemented for each platform and frequency.

A generic metadata record of the project is available via GeoNetwork at:

https://catalogue-imos.aodn.org.au/geonetwork/srv/api/records/8edf509b-1481-48fd-b9c5-b95b42247f82.

The NetCDF files are also accessible via the AODN THREDDS data server that can be accessed remotely using the OPeNDAP protocol at:

http://thredds.aodn.org.au/thredds/catalog/IMOS/SOOP/SOOP-BA/catalog.html.

A snapshot of processed NetCDF files at the time of this publication has been assigned a Digital Object Identifier (10.26198/dv5p-t593) and will be maintained in perpetuity by the AODN^77^. Readers are directed to check the AODN Portal for the latest data set.

## Technical Validation

### Routine calibration and monitoring of echosounders

In the context of echosounder calibration, it is important to note that respective $\pm X$ dB re 1 (where $X$ is a real number) change in calibration parameters ${G}_{0}$ and ${S}_{a{\rm{c}}{\rm{o}}{\rm{r}}{\rm{r}}}$ factor represents a corresponding twofold $\mp 2X$ dB re 1 m^2^ m^-3^ variation in the derived ${S}_{v}$ (Eq. 1) that would result in $\mp \left(100\left(1{0}^{(2X/10)}\right)-100\right)$% change echo-integration results (if accurate calibration parameters are not applied). In principle, properly calibrated echosounders operating at the same frequency should provide matching echo-integration results for a given sampling region. However, due to platform performance (e.g. aeration beneath the transducer), the derived data may be biased and this can be verified by an intercalibration^4,78^ experiment with two or more platforms simultaneously sailing over the same region, and later comparing the echo-integration results. In suitable circumstances, large uncertainty in the absolute calibrations and platform-specific factors can be quantified. This generic principle was applied to prioritize platforms for potential long-term data collection by comparing data quality metrics between participating platforms. As the spatio-temporal coverage of the data series improves, it will be possible to perform more direct comparisons between platforms and with an acoustic climatology of the regions.

**Fig. 7:**
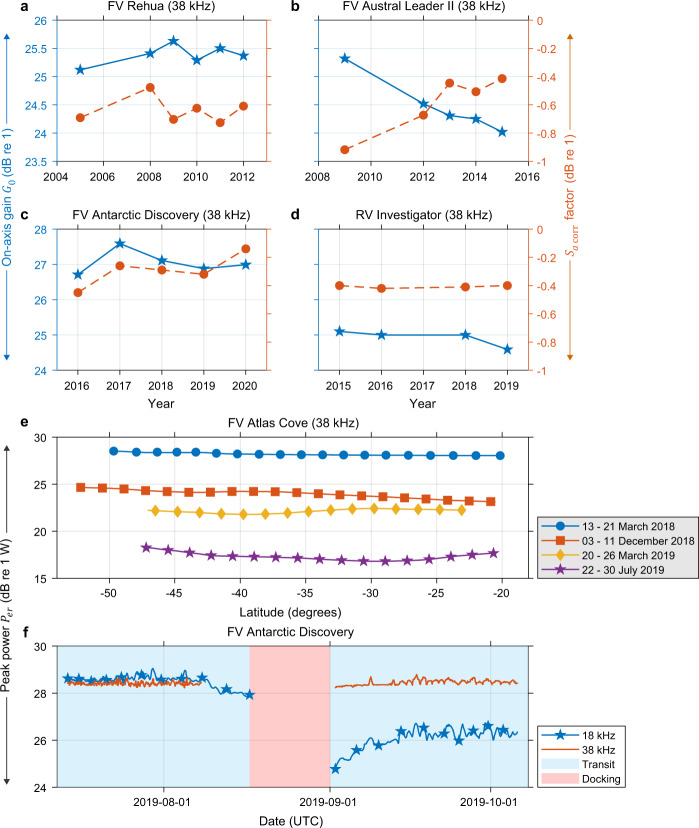
Monitoring long-term performance and stability of echosounders. Panels (**a**–**d**) show 38 kHz calibrated *G*_0_ (blue solid line) and ${S}_{a{\rm{c}}{\rm{o}}{\rm{r}}{\rm{r}}}$ factor (red dash line) for (**a**) FV Rehua (ES38-B SN30218), (**b**) FV Austral Leader II (ES38-B SN30835), (**c**) FV Antarctic Discovery (ES38-7 SN111), and (**d**) RV Investigator (ES38-B SN31167) with consistent echosounder configuration. (**e**) Spatial and temporal variations in peak power ${P}_{er}$ (measured within 0–1 m range) for FV Atlas Cove (ES38-7 SN171) over a year. (**f**) A comparison between 18 (ES-18 SN2112) and 38 kHz (ES38-7 SN111) peak power values for FV Antarctic Discovery covering 15 days docking period, highlighting the importance of routine monitoring and calibration.

Time series calibration results of selected platforms (with consistent echosounder configuration) are shown in Fig. 7 as an example to highlight repeatability and challenges with monitoring long-term performance and stability of echosounders. The FV Rehua, FV Antarctic Discovery, and RV Investigator demonstrate reasonable repeatability of 38 kHz transducer measurements with ${G}_{0}$ values varying between 25.4 ± 0.2, 27.0 ± 0.3, and 24.9 ± 0.2 dB re 1 respectively (Fig. 7a,c,d). In contrast, the FV Austral Leader II (Fig. 7b) indicates gradual degradation of system performance (possibly ageing effect) over six years with 1.3 dB re 1 decrease in calibrated ${G}_{0}$ values^79^. Keeping ${p}_{et}$, $\tau$, ${\alpha }_{a}$, ${c}_{w}$, and $\psi$ constant (Eq. 1), this performance change would result in ~44% decrease in ${S}_{v}$ data if ${G}_{0}$ and ${S}_{a{\rm{c}}{\rm{o}}{\rm{r}}{\rm{r}}}$ factor calculated in 2009 is applied for processing 2015 data sets.

Although the established sphere calibration method standardizes bioacoustic data collected by multiple participating platforms, there is a need for an additional diagnostic method to ensure echosounder performance in between routine calibrations. Along with calibration results, the peak values of instantaneous received power ${P}_{er}$ (Eq. 1) measured within 0–1 m range (i.e. ringdown zone) are used as a complementary diagnostic method to ensure stability of echosounders, noting that monitoring is not calibration. This method can highlight noticeable gradual or abrupt changes in system performance over time. For example, spatio-temporal variations in peak power for FV Atlas Cove (Fig. 7e) highlight gradual degradation of 38 kHz echosounder performance with ~11 dB re 1 W decrease in peak power values over a year, complicating data usage. In contrast, a comparison between 18 and 38 kHz peak power values for FV Antarctic Discovery (Fig. 7f) highlights an unknown abrupt change (~3 dB re 1 W) in 18 kHz echosounder performance over 15 days docking period, necessitating routine monitoring. Such performance changes (if observed) are reported back to the operator for system maintenance (Fig. 1), and juxtaposed with relevant calibration results to assess repeatability of measurements and prioritizing transects for processing.

Simmonds and MacLennan^2^ consider that in fisheries acoustics applications, properly maintained low-frequency scientific echosounders can demonstrate consistent performance within 10% in the long-term. The aim should be to develop a routine or protocol for calibration that would help achieve this accuracy consistently irrespective of the echosounder system used. But in practice, variability in echosounder on-axis sensitivity could result from a combination of factors including system electronics, data acquisition settings, SNR, environmental conditions, and density and composition of the calibration sphere^3^. The performance of an echosounder may degrade gradually or abruptly (Fig. 7e,f), and transducers are vulnerable to mechanical damage and ageing effects^80^. Therefore, it is important to quantify such changes routinely for all participating platforms to apply suitable calibration corrections required for data processing. This would further facilitate existing feedback mechanism with platform operators for subsequent system maintenance and technical inspection.

### Transducer motion correction

**Fig. 8:**
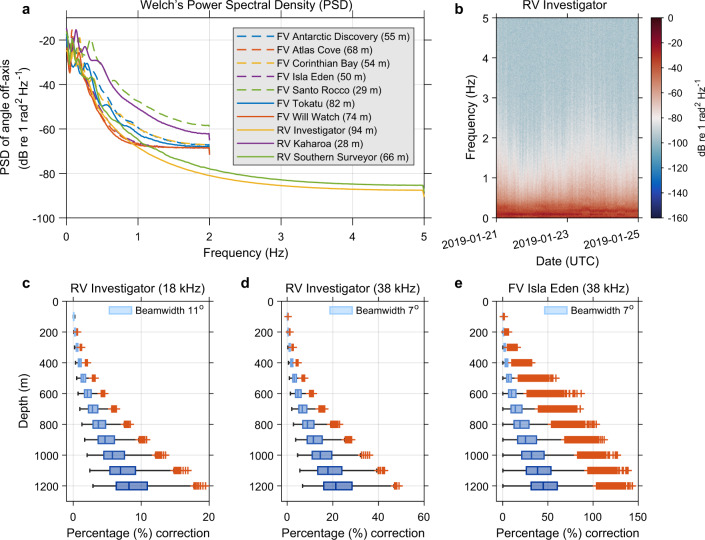
Importance of transducer motion correction. (**a**) Checking the precondition of Dunford^68^ algorithm using PSD analyses of motion data recorded by selected vessels with varying dimensions and range of weather conditions, indicating the strength of variations (energy) in pitch and roll data as a function of waveform frequency. Recorded pitch and roll data were converted from Cartesian to polar coordinate for translating platform motion as transducer angle off-axis. (**b**) Shows spectrogram analysis of example motion data recorded by RV Investigator at a sampling rate of 10 Hz, indicating temporal variations in waveform frequencies with insignificant energy contribution from rapid vessel movements above 2 Hz. Panels (**c**,**d**) display magnitudes and effects of transducer motion correction (see Table 7 for description) applied to 18 and 38 kHz calibrated raw ${s}_{v}$ data recorded onboard RV Investigator during 12–13 March 2018 in Southern Ocean, highlighting expected changes between beamwidths of transducers used. Similarly, (**e**) the magnitudes of motion correction applied to 38 kHz calibrated raw ${s}_{v}$ data recorded onboard FV Isla Eden during 06–16 June 2019 in Southern Indian Ocean is provided to highlight appreciable changes between vessel design and nature of sea state. Note the non-linear range dependent effect in all cases. In boxplots, the vertical line inside of each box is the sample median. The right and left edges of each box are the upper and lower quartiles respectively. The distance between the right and left edges is the interquartile range (IQR). Values that are more than 1.5 IQR away from the right or left of the box are outliers (red plus sign).

Transducer motion can reduce the received signal and degrade data quality substantially at long ranges depending on the sea state and platform design. For hull-mounted circular transducer, the platform motion and transducer motion can be considered synonymous^81^. Accordingly, the angular motion of platform can be used to correct for the change in orientation of transducer beam between the times of each ping, with a precondition that platform motion data (i.e. pitch and roll) need to be recorded at a sampling rate above the Nyquist rate of platform’s angular motion. The Power Spectral Density (PSD) analyses^82^ of motion data (Fig. 8a) recorded from selected platforms indicate that a minimum sampling rate of 4 Hz is generally adequate to meet this precondition and subsequent correction. Sources of error may exist in motion-corrected data if there is a large discrepancy between measured and manufacturer specified (or nominal) beamwidths of the transducer used^83^.

Owing to the magnitude of angular displacement and beamwidth values of transducers used, the effects of transducer motion can result in a non-linear range dependent ${s}_{v}$ correction. If motion correction is not applied, it can negatively bias (or underestimate) echo-integration results, where the amount of correction increases with range. The correction is greater for narrow-beam transducers and comparatively smaller for wide-beam transducers (Fig. 8c,d). The variable ‘motion_correction_factor’ (Table 7) is now being stored in NetCDF files for assessing the magnitude of transducer motion correction and recalculating calibrated raw data (if needed).

Associated global attributes (Fig. 6) ‘data_processing_motion_correction’ and ‘data_processing_motion_correction_description’ capture important metadata of transducer motion correction applied.

### Data processing filters

The quality of bioacoustic data collected from ships of opportunity sampling methods can be complex and extremely variable. If noise is not removed, it can be misinterpreted as biological signal, biasing echo-integration results. Statistical quantification of bias and error potential for data retained after filtering is challenging and beyond the scope of present study. However, selected examples of bioacoustic data with good and compromised quality are presented to demonstrate combined effectiveness of data processing filters. The application of data processing filters has considerably improved the quality of bioacoustic data and demonstrated to be robust across diverse platforms and weather conditions^5^. A caution is that there are subjective elements in ‘visually’ determining the quality of final data product after filtering, but this can be made objective to a certain extent by comparing raw and filtered echograms with metrics of data quality stored in NetCDF files.

**Fig. 9:**
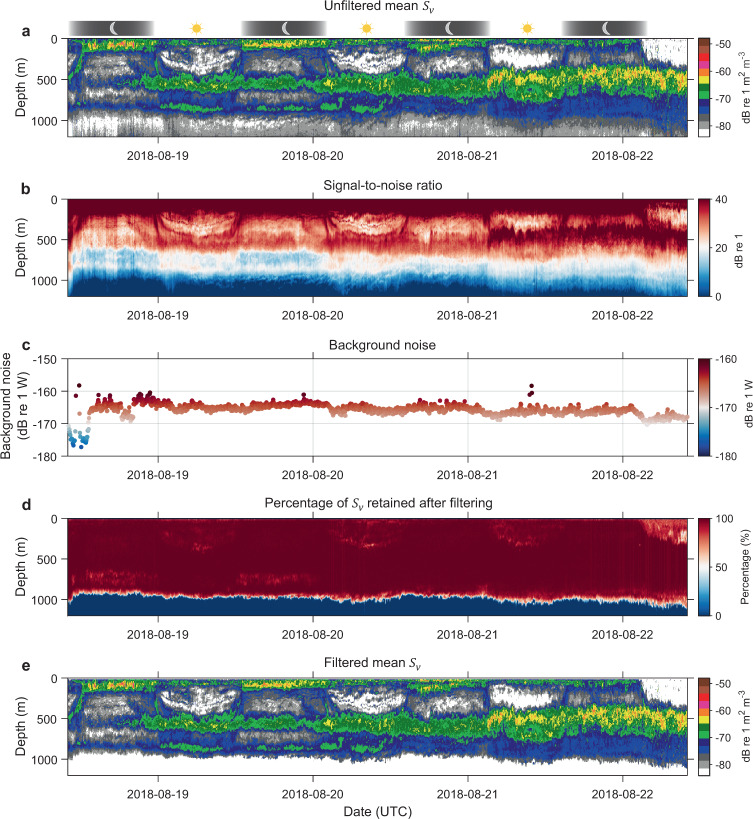
Example of good quality data comparing unfiltered and filtered echograms with metrics of data quality. The 38 kHz data was collected by FV Will Watch transiting from Mauritius to South-West Indian Ocean. (**a**) Displays calibrated and motion correction applied echogram before the application data processing filters outlined in Fig. 5. Panels (**b**,**c**) show SNR and background noise level calculated after background noise removal filter used in the filtering stage. (**d**) Depicts percentage of data retained after the filtering process. (**e**) Filtered data before applying secondary corrections for sound speed and absorption variation.

As an example, good quality data collected by FV Will Watch in the Indian Ocean is presented in Fig. 9, highlighting diel vertical migration and deep scattering layer without any apparent artefacts in the data. To broadly quantify the combined effect of data processing filters, mean difference between unfiltered and filtered echograms (i.e. difference in mean ${S}_{v}$ before and after filtering) is calculated for epipelagic, upper mesopelagic, and lower mesopelagic layers respectively, indicating 0.3 ± 0.9 (~7%), 0.1 ± 0.4 (~2%), and 0.1 ± 0.1 dB re 1 m^2^ m^-3^ (~2%) reduction in the filtered data (see Table 7 for layer description). The data quality metric SNR (Fig. 9b) in epipelagic, upper mesopelagic, and lower mesopelagic layers are 59.1 ± 4.6, 34.6 ± 3.5, and 31.5 ± 4.4 dB re 1 respectively, with mean ping-axis interval background noise level calculated as −165.6 ± 2.1 dB re 1 W (Fig. 9c). After the filtering process, approximately 98%, 98%, and 99% of ${S}_{v}$ data are retained in the epipelagic, upper mesopelagic, and lower mesopelagic layers respectively (Fig. 9d).

**Fig. 10:**
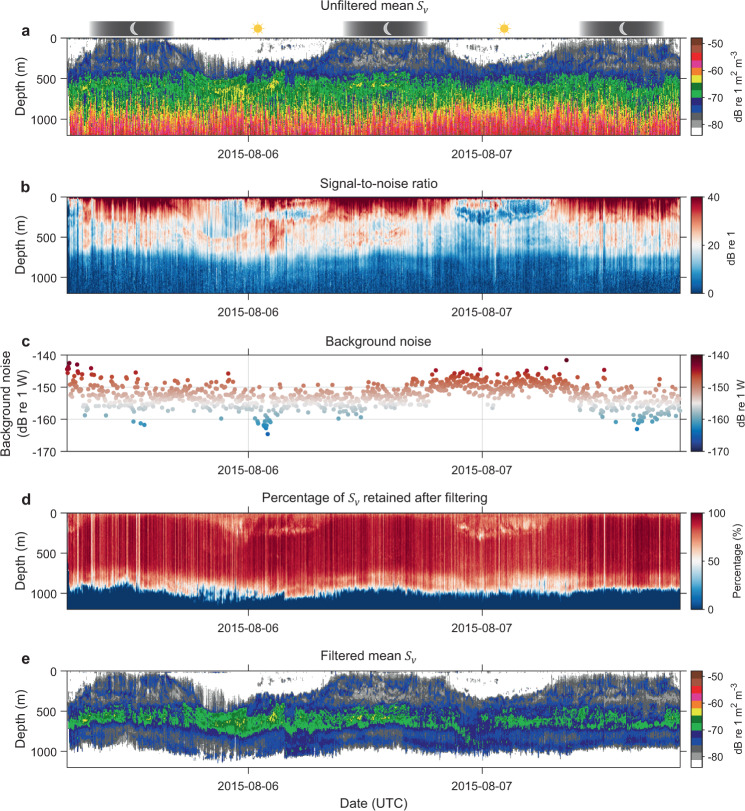
Example of compromised quality data comparing unfiltered and filtered echograms with metrics of data quality. The 38 kHz data was collected by a prospective FV San Tongariro transiting from Hobart to New Zealand in Tasman Sea. (**a**) Displays calibrated and motion correction applied echogram before the application data processing filters outlined in Fig. 5. Panels (**b**,**c**) show SNR and background noise level calculated after background noise removal filter used in the filtering stage. (**d**) Depicts percentage of data retained after the filtering process. (**e**) Filtered data before applying secondary corrections for sound speed and absorption variation.

To demonstrate the usefulness of data quality metrics, data collected by FV San Tongariro in Tasman Sea is presented in Fig. 10. This example compares raw and filtered echograms, highlighting predominant transient noise in the data amplified as a function of TVG. The mean difference between unfiltered and filtered echograms in epipelagic, upper mesopelagic, and lower mesopelagic layers respectively indicates 1.7 ± 1.9 (~48%), 1.2 ± 1.5 (~31%), and 3.6 ± 1.8 dB re 1 m^2^ m^-3^ (~129%) reduction in the filtered data, highlighting range-dependant effect of combined noise^5^ (i.e. the sum of impulse, transient, and background noise). Associated data quality metric SNR (Fig. 10b) in epipelagic, upper mesopelagic, and lower mesopelagic layers are 32.6 ± 7.9, 22.2 ± 5.3, and 17.8 ± 4.4 dB re 1 respectively, with mean ping-axis interval background noise level (Fig. 10c) calculated as −152.5 ± 3.4 dB re 1 W (note this background noise is ~13 dB re 1 W higher as compared to the good quality data presented in Fig. 9c). After the filtering process, approximately 84%, 88%, and 86% of ${S}_{v}$ data are retained in the epipelagic, upper mesopelagic, and lower mesopelagic layers respectively (Fig. 10d). The raw data quality of this transect is not satisfactory (Fig. 10a) and despite the visual appearance of filtered data, the quality metrics SNR, background noise, and percentage of ${S}_{v}$ data retained after filtering are not considered to be acceptable as compared to the other transect with high data quality (Fig. 9a).

**Fig. 11:**
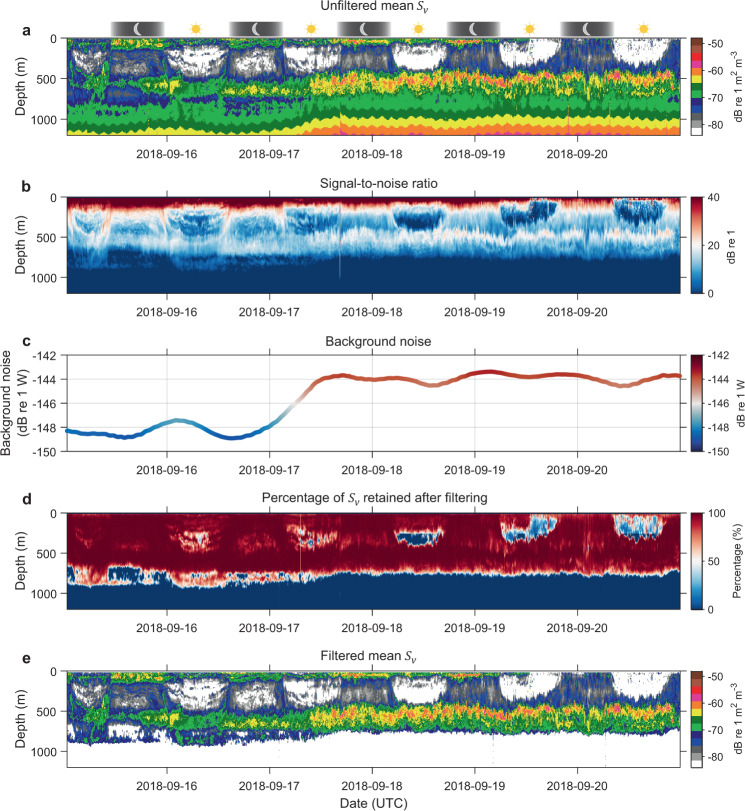
Another example of compromised quality data comparing unfiltered and filtered echograms with metrics of data quality. The 38 kHz data was collected by FV Isla Eden transiting from Mauritius to Heard Island and McDonald Islands in Southern Indian Ocean. (**a**) Displays calibrated and motion correction applied echogram before the application data processing filters outlined in Fig. 5. Panels (**b**,**c**) show SNR and ‘smoothed’ background noise level calculated after background noise removal filter used in the filtering stage. Note that the triangle wave error present in background noise level was smoothed for comparison purposes. For this transect, the error was not averaged to zero over the 1 km horizontal distance, possibly due to slow ping rate achieved. (**d**) Depicts percentage of data retained after the filtering process. (**e**) Filtered data before applying secondary corrections for sound speed and absorption variation.

Similarly, data acquired by FV Isla Eden is presented in Fig. 11, highlighting an abrupt (~5 dB re 1 W) change in the background noise level over 24 hours, presumably indicating electrical interference and electrical noise in the echosounder. The mean difference between unfiltered and filtered echograms in epipelagic, upper mesopelagic, and lower mesopelagic layers respectively indicates 1.0 ± 2.1 (~25%), 1.0 ± 1.6 (~25%), and 1.4 ± 1.2 dB re 1 m^2^ m^-3^ (~38%) reduction in the filtered data, predominantly highlighting range-dependant effect of background noise. Related data quality metric SNR (Fig. 11b) in epipelagic, upper mesopelagic, and lower mesopelagic layers are 37.5 ± 8.2, 12.9 ± 4.2, and 12.9 ± 2.3 dB re 1 respectively, with mean ping-axis interval background noise level (Fig. 11c) calculated as −145.5 ± 2.1 dB re 1 W (note this background noise is ~20 dB re 1 W higher as compared to the good quality data presented in Fig. 9c). After the filtering process, approximately 89%, 85%, and 82% of ${S}_{v}$ data are retained in the epipelagic, upper mesopelagic, and lower mesopelagic layers respectively (Fig. 11d).

These examples (Figs. 10 and 11) suggest that caution is needed while reviewing a final data product where filtering and subsequent resampling to predefined NetCDF file resolution (1 km horizontal distance and 10 m vertical depth) may produce a visually clean echogram without any apparent artefacts, but potentially removed significant biological signal and/or retained noise in the process. Accordingly, we have not posted these two transects to the AODN, and similar data sets from other platforms with compromised data quality are archived locally. Storing metrics of data quality in NetCDF files is intended for assisting users to make an independent assessment of data quality based on the examples demonstrated here.

### Secondary corrections for sound speed and absorption variation

**Fig. 12:**
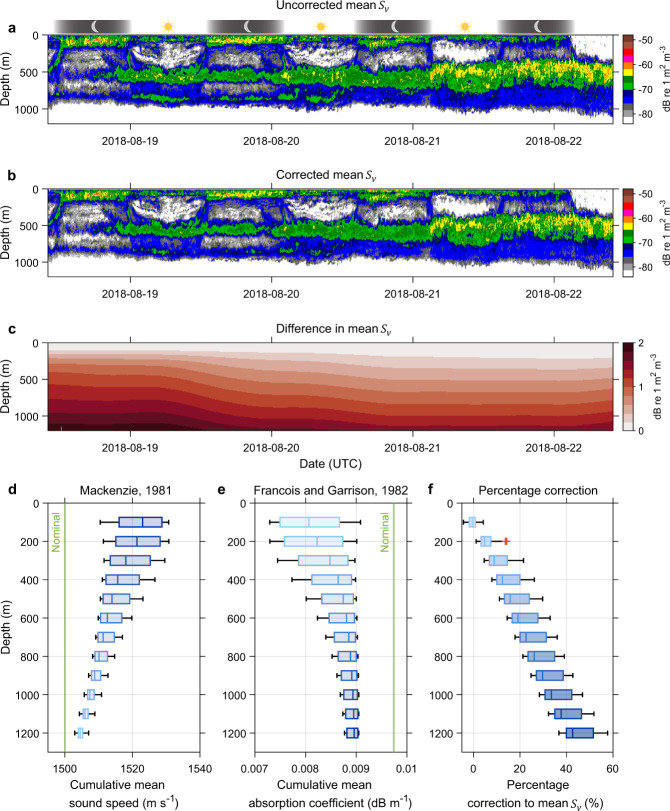
Secondary corrections for sound speed and absorption variation. The transect presented in Fig. 9 is used to highlight the nature of corrections. (**a**) Filtered but uncorrected echogram before applying secondary corrections. (**b**) Shows filtered and corrected echogram after applying secondary corrections with (**c**) result correction matrix. Bottom panels show the (**d**) cumulative mean sound speed and (**e**) absorption coefficient values used for the correction (Eq. 12), highlighting the nature of (**f**) range-dependant percentage correction applied to the final data product before depth interpolation. The nominal sound speed and absorption coefficient values used for the correction are highlighted as vertical green lines. In boxplots, the vertical line inside of each box is the sample median. The right and left edges of each box are the upper and lower quartiles respectively. The distance between the right and left edges is the interquartile range (IQR). Values that are more than 1.5 IQR away from the right or left of the box are outliers (red plus sign).

The difference between variable ‘uncorrected_Sv’ (i.e. filtered data before secondary corrections) and ‘abs_corrected_sv’ (i.e. same data after secondary corrections but before depth interpolation) is calculated (uncorrected_Sv—abs_corrected_sv) to demonstrate the effect of secondary corrections (Fig. 12). This step introduces a range-dependent correction (Fig. 12f) that can differ substantially based on the equation used for calculating sound absorption in seawater (see Fig. 5 in Doonan, *et al*.^84^ for a comparison between two commonly used equations for absorption calculation. Note that the range-dependant percentage correction to the data can differ up to 45% between Doonan, *et al*.^84^ and Francois and Garrison^71^ for a 38 kHz data at 1000 m depth).

The processed bioacoustic data sets are consistently corrected based on Mackenzie^70^ sound speed and Francois and Garrison^71^ absorption equations following the recommendations by Simmonds and MacLennan^2^ until more evidence is available to select another formula. Macaulay*, et al*.^85^ conducted field measurements of acoustic absorption in seawater from 38 to 360 kHz, indicating consistent results with Francois and Garrison^71^ equation for frequencies of 200 kHz and lower. Macaulay*, et al*.^85^ observed a significant difference around 333 kHz, indicating that Francois and Garrison^71^ equation is incorrect for some input parameters (note that 333 kHz data is not processed under IMOS Bioacoustics sub-Facility).

It is important to note that the percentage correction shown in Fig. 12 is applicable to the example transect only that depends on the nominal sound speed and absorption values used during initial processing and echo-integration (Eq. 1). Other transects (e.g. Southern Ocean) have different correction factors that are related to regional changes in temperature and salinity values.

The key intermediate variable ‘uncorrected_Sv’ is stored in NetCDF files for recalculating secondary corrections using other equations or data sources (if needed). The equation used for sound absorption calculation is documented in the global attribute section of a NetCDF file as ‘data_processing_absorption_description’ and ‘history’. Similarly, the equation used for sound speed calculation is captured in the global attributes ‘data_processing_soundspeed_description’ and ‘history’.

## Usage Notes

When interpreting bioacoustic data it is important to understand the corrections applied at each processing step particularly calibration, transducer motion correction, data filtering, and secondary corrections for sound speed and absorption variation (Fig. 5). The transducer motion and secondary corrections are range-dependant that can greatly influence the lower mesopelagic layer derived metrics. Our goal is to keep minimum updates to data processing steps and data records so that the database remains consistent and comparable.

### Measurement uncertainty

The widely used Simrad EK60 echosounder is now discontinued and replaced by the Simrad EK80. A recent comparison study^86^ highlighted that EK80 raw power measurements were 3–12% lower as compared to EK60, affecting weak scatterer and/or long-range acoustic observations due to nonlinear amplification of low-power signals by the EK60. Presently the users need to correct the data for this bias, and we are in the process of providing a correction update to the data sets with associated metadata. In addition to calibration and unknown methodological uncertainties, this new measurement uncertainty highlights the ongoing challenges in maintaining a diverse data series, and the need for storing fundamental echosounder measurement (i.e. received echo power) and appropriate metadata to enable unforeseen corrections in the future as needed.

### Challenges with biomass estimation

Ships of opportunity bioacoustic sampling methods have clear advantages as well as limitations. Their usefulness in resource assessment, ecosystem monitoring, and cost-effective mapping of mesopelagic communities at regional and global scales is established with diverse acoustic-based indicators and metrics, but credible conversion of bioacoustic data (${s}_{v}$) to open ocean fish biomass is a multi-step procedure and require lowest degree of bias.

For example, the processed ${s}_{v}$ values are vertically integrated over a measurement range (${r}_{1}$ to ${r}_{2}$) to calculate area backscattering coefficient ${s}_{a}$ (m^2^ m^−2^) along a transect. Scatterer areal density (number m^−2^) i.e. the number of organisms (e.g. fish) within the measurement range is calculated by dividing ${s}_{a}$ by the backscattering cross-section ${\sigma }_{bs}$ (m^2^) of a representative single fish. Biomass of fish (kg m^−2^) can be estimated by multiplying this scatterer areal density by the weight $W$ (kg) of a single fish. This requires separation of bioacoustic data by species composition, location, and ${\sigma }_{bs}$ distribution. Mean weight can be derived from observed weights (using nets) or length to weight regression. Similarly, ${\sigma }_{bs}$ are obtained from *in situ* measurements and/or ${\sigma }_{bs}$to length regressions^2^. Biomass calculations from these equations will be biased if the weight and target strength $TS$ [$10\,{{\rm{\log }}}_{10}({\sigma }_{bs})$, dB re 1 m^2^] of the organism are uncertain (assuming accurate calibration and echosounder linearity). For that reason, *in situ* and/or modelled $TS$ must be calculated with the goal of obtaining a representative distribution.

Credible estimation of biomass using a vessel-based echosounder is very difficult for the highly diverse mesopelagic community, where gas inclusions may present in many organisms (depending on the region) that can cause frequency- and depth-dependent resonance scattering^87^, dominating the received signal. Multiple methods of ecosystem models, net capture, acoustic backscattering models, and *in situ* profiling acoustic optical systems^19,88^ are needed to provide the necessary information to convert basin-scale bioacoustic data into specific biological metrics such as species-specific biomass^61^.

### Reading the data

Generated data are stored in NetCDF files that can be readily imported into a wide variety of cross-platform software programs and programming languages. A custom MATLAB® function ‘viz_sv’ to read and visualize NetCDF files conforming to the conventions described in this data descriptor can be downloaded from the IMOS Bioacoustics sub-Facility web site http://imos.org.au/facilities/shipsofopportunity/bioacoustic or GitHub https://github.com/CSIRO-Acoustics/Visualize-IMOS-Bioacoustics-data.

### Terms of use

All NetCDF files are released under the license Creative Commons Attribution 4.0 International (CC-BY 4.0, https://creativecommons.org/licenses/by/4.0/). Any users of IMOS data are required to clearly acknowledge the source of the material in the format: “Data was sourced from Australia’s Integrated Marine Observing System (IMOS) – IMOS is enabled by the National Collaborative Research Infrastructure Strategy (NCRIS). It is operated by a consortium of institutions as an unincorporated joint venture, with the University of Tasmania as Lead Agent.”

## Data Availability

Echosounder raw data files are recorded in proprietary formats that typically require dedicated commercial or open-source acoustic processing software for visualization and processing. The custom *Java* software tool ‘*basoop.jar*’ used for incoming data registration and management, along with the MATLAB® GUI used for controlling data processing steps in Echoview® and NetCDF file creation can be obtained at: https://github.com/CSIRO-Acoustics/IMOS-Bioacoustics. The MATLAB® codes used for generating relevant figures are available at: https://github.com/CSIRO-Acoustics/Publications/tree/main/Scientific_Data/Data_Descriptor.
